# Parental religiosity is associated with changes in youth functional network organization and cognitive performance in early adolescence

**DOI:** 10.1038/s41598-022-22299-6

**Published:** 2022-10-15

**Authors:** Skylar J. Brooks, Luyao Tian, Sean M. Parks, Catherine Stamoulis

**Affiliations:** 1 Division of Adolescent Medicine, Department of Pediatrics, Boston Children’s Hospital, Boston, MA USA; 2 Massachusetts Institution of Technology, Cambridge, MA USA; 3 Harvard Medical School, Boston, MA USA; 4 Department of Pediatrics, Boston Children’s Hospital, 300 Longwood Avenue, Boston, MA 02115 USA

**Keywords:** Neuroscience, Cognitive neuroscience, Computational neuroscience, Neural circuits

## Abstract

Parental religious beliefs and practices (religiosity) may have profound effects on youth, especially in neurodevelopmentally complex periods such as adolescence. In n = 5566 children (median age = 120.0 months; 52.1% females; 71.2% with religious affiliation) from the Adolescent Brain Cognitive Development study, relationships between parental religiosity and non-religious beliefs on family values (data on youth beliefs were not available), topological properties of youth resting-state brain networks, and executive function, inhibitory control, and cognitive flexibility were investigated. Lower caregiver education and family income were associated with stronger parental beliefs (p < 0.01). Strength of both belief types was correlated with lower efficiency, community structure, and robustness of frontoparietal control, temporoparietal, and dorsal attention networks (p < 0.05), and lower Matrix Reasoning scores. Stronger religious beliefs were negatively associated (directly and indirectly) with multiscale properties of salience and default-mode networks, and lower Flanker and Dimensional Card Sort scores, but positively associated with properties of the precuneus. Overall, these effects were small (Cohen’s d ~ 0.2 to ~ 0.4). Overlapping neuromodulatory and cognitive effects of parental beliefs suggest that early adolescents may perceive religious beliefs partly as context-independent rules on expected behavior. However, religious beliefs may also differentially affect cognitive flexibility, attention, and inhibitory control and their neural substrates.

## Introduction

Environmental factors associated with family culture may have profound effects on the developing brain. For families/caregivers who believe in a higher power, spirituality and/or religion and related practices (collectively referred to as religiosity) are often an important part of the youth’s cultural environment, but their effects on developing brain circuits and high-level cognitive processes that continue to maturate until adulthood remain elusive. In the last two decades, a significant interest in the connection between religion and brain has led to the establishment of the rapidly growing, multidisciplinary and complex field of neurotheology^1^, which specifically focuses on the neural basis of religiosity^2–5^. However, given a constellation of correlated factors that affect the brain and change over time, decoupling and quantifying the impact of religiosity on neural circuits is challenging^6,7^, particularly during development.

Prior work on the relationship between brain and religion has focused mostly on adults, is heterogeneous and also relatively limited, compared to cognitive/behavioral studies on religiosity^8–15^. Also, experimental paradigms vary substantially across studies, making it difficult to integrate findings into a unified understanding of the relationship between religiosity, brain and behavior^4,7,16–22^. Despite earlier controversial hypotheses of a ‘God center’ in the brain^23,24^, inspired by studies linking religious hallucinations to the temporal lobes and the limbic system^24–31^, the neurotheology field is now in some agreement that spirituality/religiosity may be associated with distributed brain regions^32–35^. Neuroimaging studies have linked religiosity to activation changes in parietal, temporal, parahippocampal, and cerebellar regions, multiple gyri in the frontal, temporoparietal and occipital lobes (predominantly in the right hemisphere), and the insula, caudate nucleus, anterior cingulate cortex (ACC), precuneus, and medial prefrontal cortex^17,19,36–41^. A recent study also identified the periaqueductal gray (PAG)—a brainstem structure, as a critical hub in the network involved in spiritual experiences^34^. These findings are further supported by neuroanatomical studies showing that higher religiosity/spirituality is associated with increased thickness of bilateral parietal and occipital cortical areas, right mesial frontal cortex, and left cuneus and precuneus^42^. However, other studies have found no clear association between religiosity and structural brain changes, suggesting that it may instead impact functional neural circuits^43^, and in some cases no statistical associations between religiosity and functional activations (or cognitive performance), specifically in tasks related to conflict processing^44^.

Despite insights into the neural correlates of religiosity in adults, its effects on the developing brain are unclear. Yet, with 70% of Americans considering religion at least somewhat important in their lives and 40% considering it very important^45^, many children grow up in families that believe in the divine and engage in religious practices. Epidemiological studies have shown that regular attendance of religious services, prayer, and/or meditation in adolescence are associated with better physical, psychological, and mental health outcomes and lower likelihood of risk behaviors in young adulthood^46,47^. A cross-sectional study based on a nationally representative sample of primary school-age children showed that religion may have an overall positive effect on social skills and psychological outcomes^48^. However, a longitudinal investigation of the same sample (over 10,000 children, from kindergarten to third grade) showed that, despite positive effects in some domains, parental religiosity is also associated with adverse effects in scholastic performance in mathematics and science^49^. Findings from other studies also suggest that parental religiosity may have positive and negative effects on child development^50^, but none of these studies investigated the neural correlates of religiosity in children.

Typically developing neural circuits are highly heterogeneous. Thus, for generalizability, the impact of religiosity needs to be studied in large samples. This is especially important in adolescence, a period of significant social growth, during which the parent-youth relationship and influence of parental beliefs change substantially^51,52^, and the youth’s social environment expands beyond the family^53,54^. These changes may play a significant role in maintaining, questioning, or rejecting religious beliefs^55^, but may also shape the unique development of brain circuits^56–58^. Thus, the relationships between youth religiosity, functional circuits and cognitive function (particularly flexibility, control) are likely bidirectional^59^.

To address the sample size limitations of many neurotheological studies and lack of direct brain activity measurements in epidemiological or behavioral studies, we investigated the effects of parental religiosity on developing functional neural circuits and their cognitive correlates in a large early adolescent cohort. Resting-state fMRI and neurocognitive assessments^60^ from n = 5566 early adolescents in the Adolescent Brain Cognitive Development (ABCD) study^61^, and associated survey data on parental religious and family values (i.e., religion-independent) beliefs were analyzed. We hypothesized that parental religiosity modulates the topological organization of functional brain networks in youth and the cognitive processes they support, in distinct ways compared to religion-independent beliefs. In addition, parental religiosity impacts youth cognitive performance both directly and indirectly through its mediating effects on the underlying neural circuitry, and also moderates the relationship between functional network organization and cognitive performance. We focused specifically on cognitive tasks measuring executive function and control, attention, cognitive flexibility, and problem solving ability, i.e., processes that continue to evolve during adolescence. Statistical mediation and moderation models were developed to test the hypothesized relationships, and a split-sample approach was used for model validation.

## Results

We studied n = 5566 early adolescents, 2669 males (47.95%) and 2896 females (52.08%), median age = 120.0 months (IQR = 13.0 months). About half of participants (n = 3014; 54.15%) were in pre- or early puberty and a quarter (n = 1427; 25.64%) in mid-puberty. Racial and ethnic distributions in our sample (67.09% white and 79.21% non-Hispanic) reflected those of the ABCD cohort, which is similarly unbalanced in terms of race and ethnicity. Median yearly family income range was $75,000–99,000, about a quarter of families (n = 1363; 24.49%) had a combined income < $50,000/year, and n = 2297 (41.27%) had an income ≥ $100,000/year. Over half of caregivers had at least a bachelor’s degree (n = 3125; 56.15%). Youth and parent/family demographic characteristics are summarized in Table 1.

**Table 1:** Youth/family demographics.

	N (%)
**Sex**	
Male	2669 (47.95)
Female	2896 (52.03)
Missing	1 (0.02)
**Race**	
White	3734 (67.09)
Black	1008 (18.11)
Asian	346 (6.22)
American Indian/Alaska Native	121 (2.17)
Hawaiian/Pacific Islander	24 (0.43)
Other	256 (4.60)
Missing	77 (1.38)
**Ethnicity**	
Hispanic	1097 (19.71)
Non-Hispanic	4409 (79.21)
Missing	60 (1.08)
**Yearly family income ($)**	
< 5000	145 (2.61)
5000–24,999	480 (8.62)
25,000–49,999	738 (13.26)
50,000–99,999	1496 (26.88)
100,000–199,999	1653 (29.70)
≥ 200,000	644 (11.56)
Missing	410 (7.37)
**Primary caregiver education**	
Advanced degree*	1408 (25.30)
Bachelor’s degree	1717 (30.85)
Associate degree	684 (12.29)
Some college	914 (16.42)
High School	538 (9.67)
Did not graduate high school	300 (5.39)
Missing	5 (0.08)

*Includes master’s professional (MD, JD, etc.) and doctoral degrees.

### Religious preferences, beliefs, and practices

Over 70% of primary caregivers indicated that their child had a religious preference, with 1028 (18.46%) identifying as Roman Catholic, 1043 (11.33%) Protestant, 632 (11.35%) Mormon, 764 (13.73%) as one of the other faiths/denominations in the survey, 76 (1.37%) with a religious preference not listed in the survey, 256 (4.60%) atheist or agnostic, and 1119 (20.10%) with no particular religious preference. There were no statistically significant sex differences in the distribution of responses (p = 0.26), but there was a statistical difference between white and non-white (p < 0.01) and Hispanic vs non-Hispanic (p = 0.02). Compared to non-white participants, 2.5 times as many white participants identified as mainline Protestant, twice as many as Roman Catholic, and 6 times as Mormon. Three times as many Hispanics identified as Roman Catholic compared to non-Hispanics, and twice as many non-Hispanics identified as Protestant.

More than half of caregivers strongly believed in the power of faith in God and importance of prayer and religion (Table 2). Over 60.0% reported that religion was important in their child’s life. About 35.0% of participants attended religious services at least weekly, and over 25.0% never attended services (Table 3). There was a significant positive association between the strength of parental religious beliefs and youth frequency of service attendance and importance of religion in their life (p < 0.01). The strength of religious beliefs and frequency of practices were positively associated with the strength of all non-religious beliefs (p < 0.01). Lower parental education and/or family income was correlated with strength of both types of beliefs (p < 0.01).

**Table 2:** Distribution of responses to questions related to religious beliefs in the Mexican American Cultural Values Scale (MACVS). Percentages are out of n = 5566 participants.

Mexican American Cultural Values Scale (MACV): Religious beliefs N (%)					
	Not at all	A little	Somewhat	Very much	Completely
One's belief in God gives inner strength and meaning to life	662 (11.89)	463 (8.32)	946 (17.00)	1397 (25.10)	2097 (37.68)
God is first; family is second	1849 (33.22)	518 (9.31)	808 (14.52)	857 (15.40)	1533 (27.54)
Parents should teach their children how to pray	1048 (18.83)	667 (11.98)	779 (14.00)	1149 (20.64)	1922 (34.53)
If everything is taken away, one still has their faith in God	988 (17.75)	582 (10.46)	656 (11.79)	1135 (20.39)	32,204 (9.60)
It is important to thank God every day for all one has	1085 (19.49)	556 (9.99)	633 (11.37)	1014 (18.22)	2277 (40.91)
It is important to follow the word of God	1215 (21.83)	583 (10.47)	867 (15.58)	1152 (20.70)	1748 (31.40)
Religion should be an important part of one’s life	1150 (20.66)	708 (12.72)	1088 (19.55)	1152 (20.70)	1467 (26.36)
Missing data	1 (0.02)

**Table 3:** Distribution of responses to questions in the Parent Demographic Survey (PDEM) related to religion. Percentages are out of n = 5566 participants.

	Parent Demographic Survey (PDEM) N (%)					
	No preference//agnostic/atheist		Some preference		Missing	
What is the child’s religious preference?	1375 (24.70)		3961 (71.16)		230 (4.13)	

	Not at all	Not very	Somewhat	Very much	Missing	
How important are your child’s religious/spiritual beliefs in his/her daily life?	969 (17.41)	845 (15.18)	1613 (28.98)	1974 (35.47)	165 (2.96)	

	Never	< Once a month	1–3 times/month	Once a week	> Once a week	Missing
How often does your child attend religious services?	1492 (26.81)	1242 (22.31)	807 (14.50)	1538 (27.63)	384 (6.90)	103 (1.85)

### Direct effects of religious and religion-independent beliefs on connectome topology and cognitive function

#### Associations between religious beliefs/practices and cognitive task performance

**Figure 1:**
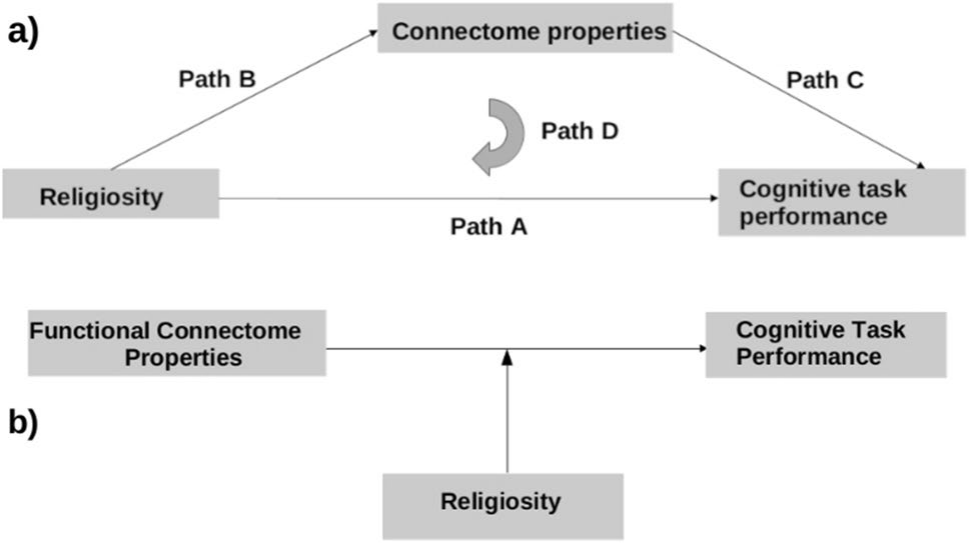
(**a**) Diagram of statistical (mediation) model assessing the direct (path A) and indirect (through modulation of connectome properties; Path B) effects of religiosity on performance in higher-level cognitive tasks. Path D represents the full model, which includes brain connectome properties as the mediator. (**b**) Diagram of moderating effects of religiosity on the relationship between connectome organization and cognitive task performance.

Direct relationships between religiosity and task scores were first assessed (Path A, Fig. 1a). Median age-corrected Flanker task score was 97.0 (IQR = 19.0). Flanker scores were negatively associated with the strength of beliefs that *God is first, family is second* (p < 0.01), *parents should teach their children how to pray* (p < 0.01), *if everything is taken away, one still has their faith in God* (p = 0.02), *it is important to thank God every day and follow the word of God* (p < 0.01), and *religion should be an important part of one’s life* (p < 0.01). The child’s religious affiliation and practices were not statistically associated with Flanker scores (p ≥ 0.16). In addition, there were no significant associations between performance in the *Cash Choice* task and any measures of religiosity (p > 0.49). Median age-corrected Card Sort task score 94.0 (IQR = 23.0). Scores were negatively associated with the strength of beliefs *parents should teach their children how to pray*, and *everything is taken away, one still has their faith in God (p* = *0.04)*, but no other religiosity measures (p > 0.07). Median *Matrix Reasoning* scaled score was 10.0 (IQR = 4.0). Scores were negatively associated with the strength of all religious beliefs (Table S1; p ≤ 0.04), but not religious practices (p > 0.21). In all models for Path A, family income was positively associated with task scores (p < 0.01). Family size was nonsignificant (p > 0.10). Importance of family togetherness was nonsignificant in models for Flanker or Card Sort scores (p > 0.07), but negatively associated with willingness to wait for a higher reward in Cash Choice (p < 0.01), and Matrix Reasoning scores (p <0.03). Based on split-sample validation, models had good fit and predictive power [CV(RMSE) ≤ 0.14].

#### Associations between religion-independent beliefs and cognitive task performance

About 20% of primary caregivers strongly believed that children should always do things to make their parents happy, while over 40% somewhat believed that. Over 50% strongly believed that children should be taught to always be good because they represent the family, and over 70% believed that it is important to work hard and do one’s best because this work reflects on the family (Table 4). There were no significant associations between the strength of these beliefs and performance in the Flanker, Cash Choice, or Card Sort task (p ≥ 0.10). There was a negative association between the strength of the belief that children should always do things to make their parents happy and Matrix Reasoning scores (p < 0.01). In the split-sample validation, this model had relatively good fit and predictive power [CV(RMSE) < 0.20]. Parental beliefs that were statistically associated with task performance are summarized in Table S1.

**Table 4:** Distribution of caregiver responses to questions on beliefs related to children’s behavior towards the family (unrelated to religion), using the Mexican American Cultural Values Scale (MACVS). Percentages are out of all n = 5566 participants.

Mexican American Cultural Values Scale (MACV): Beliefs related to children’s expected behavior towards the family (unrelated to religion) N (%)					
	Not at all	A little	Somewhat	Very much	Completely
Children should always do things to make their parents happy	919 (16.51)	1343 (24.13)	2308 (41.47)	691 (12.41)	304 (5.46)
Children should be taught to always be good because they represent the family	380 (6.83)	691 (12.41)	1696 (30.47)	1712 (30.76)	1086 (19.51)
It is important to work hard and do one's best because this work reflects on the family	288 (5.17)	521 (9.36)	1731 (31.10)	1739 (31.24)	1286 (39.27)
Missing data	1 (0.02)				

#### Associations between religious beliefs/practices and brain network properties

The relationship between religiosity and network properties (Path B, Fig. 1a) was assessed at three spatial scales. No significant associations between religious beliefs or practices and whole-brain network properties were estimated (p > 0.06).

#### Individual networks

The strength of the belief that *God is first, family is second* was negatively associated with multiple topological properties, including efficiency, global clustering, within-network connectivity, robustness and/or stability of the left tempo-parietal network (p < 0.05), the right temporoparietal network (p ≤ 0.02), the left salience/ventral attention network (p ≤ 0.03), the left dorsal attention network (p < 0.04), and the right frontoparietal control network (p = 0.04). Effect sizes were small (Cohen’s d ~ 0.20 to 0.23). Model parameters are summarized in Table 5. The strength of the belief that ‘*parents should teach their children how to pray*’ was negatively associated with multiple properties of the right temporoparietal network (p < 0.05), and similarly the strength of the belief that ‘*religion should be an important part of one’s life*’ was negatively associated with topological robustness and stability of the left dorsal attention networks (p = 0.03). No other significant associations were identified (p > 0.10). Again, effect sizes were smal (Cohen’s d ~ 0.18 to 0.26). Model parameters are summarized in Table 6. All networks impacted by religious beliefs are summarized in Table S1. In secondary analyses based on a set of structural equation models, the mediating effect of youth religiosity (the latent estimated variable) on the relationship between the strength of parental religious beliefs and network or node properties was nonsignificant (p ≥ 0.12).

**Table 5:** Parameters of models assessing the relationship between the belief that ‘*God is first, family is second’* and individual network properties. Reported p-values have been adjusted for FDR and beta coefficients have been standardized. Median connectivity within the network (in) and between a network’s nodes and the rest of the brain (out) are also reported. Across properties, effect sizes (Cohen’s d) were calculated with strength of belief = 1 (not at all) as the reference, with adjustments for unequal sample sizes. A range of effect sizes across properties, and 95% confidence intervals (CI) are provided.

Network	Model parameter	Efficiency	Global clustering	Median Conn (in)	Median Conn (out)	Robustness	Topological stability
**Belief: God is first, family is second**							
Left hemisphere							
Salience/ventral attention	Beta	− 0.035	–	–	–	− 0.039	− 0.039
	p-value	0.030	–	–	–	0.028	0.028
	Effect size (Cohen’s d)	0.197–0.218 (95% CI 0.129–0.276)					
Dorsal attention	Beta	–	–	− 0.041	–	− 0.035	− 0.036
	p-value	–	–	0.038	–	0.038	0.038
	Effect size (Cohen’s d)	0.194–0.224 (95% CI 0.126–0.292)					
Temporoparietal	Beta	− 0.040	− 0.042	–	–	–	–
	p-value	0.050	0.050	–	–	–	–
	Effect size (Cohen’s d)	0.113–0.116 (95% CI 0.095–0.184)					
Right hemisphere							
Frontoparietal control	Beta	− 0.034	− 0.036	–	–	− 0.036	− 0.035
	p-value	0.040	0.040	–	–	0.040	0.040
	Effect size (Cohen’s d)	0.206–0.229 (95% CI 0.143–0.297)					
Temporoparietal	Beta	− 0.039	− 0.045	− 0.038	–	− 0.044	− 0.044
	p-value	0.020	0.011	0.022	–	0.011	0.011
	Effect size (Cohen’s d)	0.143–0. 225 (95% CI 0.101–0.293)

**Table 6:** Parameters of models assessing the relationship between the belief that *‘parents should teach their children how to pray’* and ‘*religion should be an important part of one’s life’* and individual network properties. Across properties, effect sizes (Cohen’s d) were calculated with strength of belief = 1 (not at all) as the reference, with adjustments for unequal sample sizes. A range of effect sizes across properties, and 95% confidence intervals (CI) are provided.

Network	Model parameter	Efficiency	Global clustering	Median Conn (in)	Median Conn (out)	Robustness	Topological stability
**Belief: Parents should teach their children how to pray**							
Right hemisphere							
Temporoparietal	Beta	–	− 0.039	–	–	− 0.037	− 0.036
	p-value	–	0.049	–	–	0.049	0.049
	Effect size (Cohen’s d)	0.178–0.263 (95% CI 0.135–0.337)					
**Belief: Religion should be an important part of one’s life**							
Left hemisphere							
Dorsal attention	Beta	–	–	–	–	− 0.038	− 0.037
	p-value	–	–	–	–	0.031	0.031
	Effect size (Cohen’s d)	0.207–0.246 (95% CI 0.130–0.323)					

#### Individual regions (node-specific properties)

**Figure 2:**
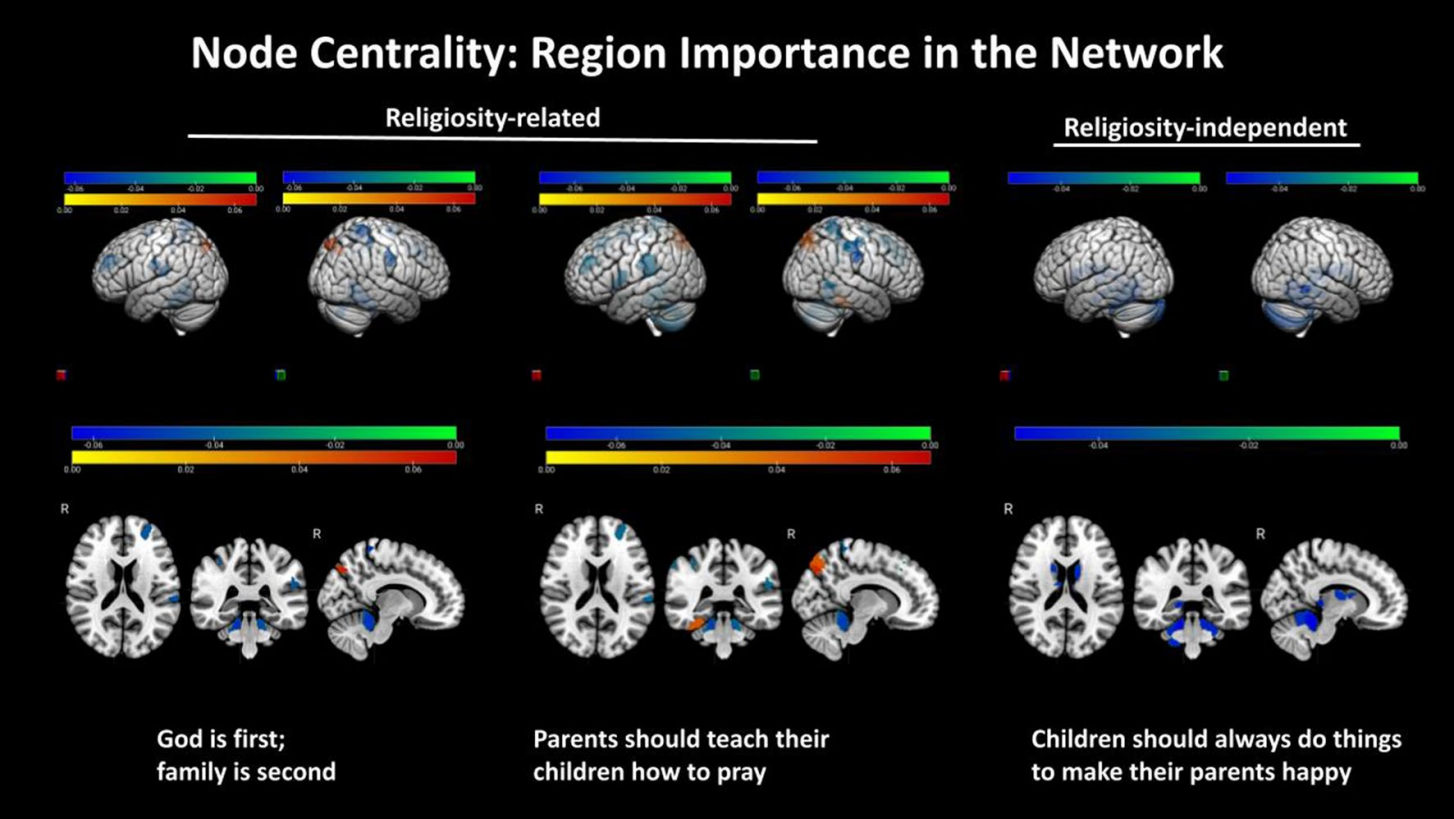
Significant positive and negative associations were estimated, between strength of religious (God is first, family is second, and parents should teach their children how to pray), one religion-independent belief (children should always do things to make their parent happy), and node centrality (regional importance in a network). The colorbars represent the range of positive (yellow–red) and negative (green–blue) standardized regression coefficient values in statistical models that assessed these effects. Two and three-dimensional views of both hemispheres are shown.

**Figure 3:**
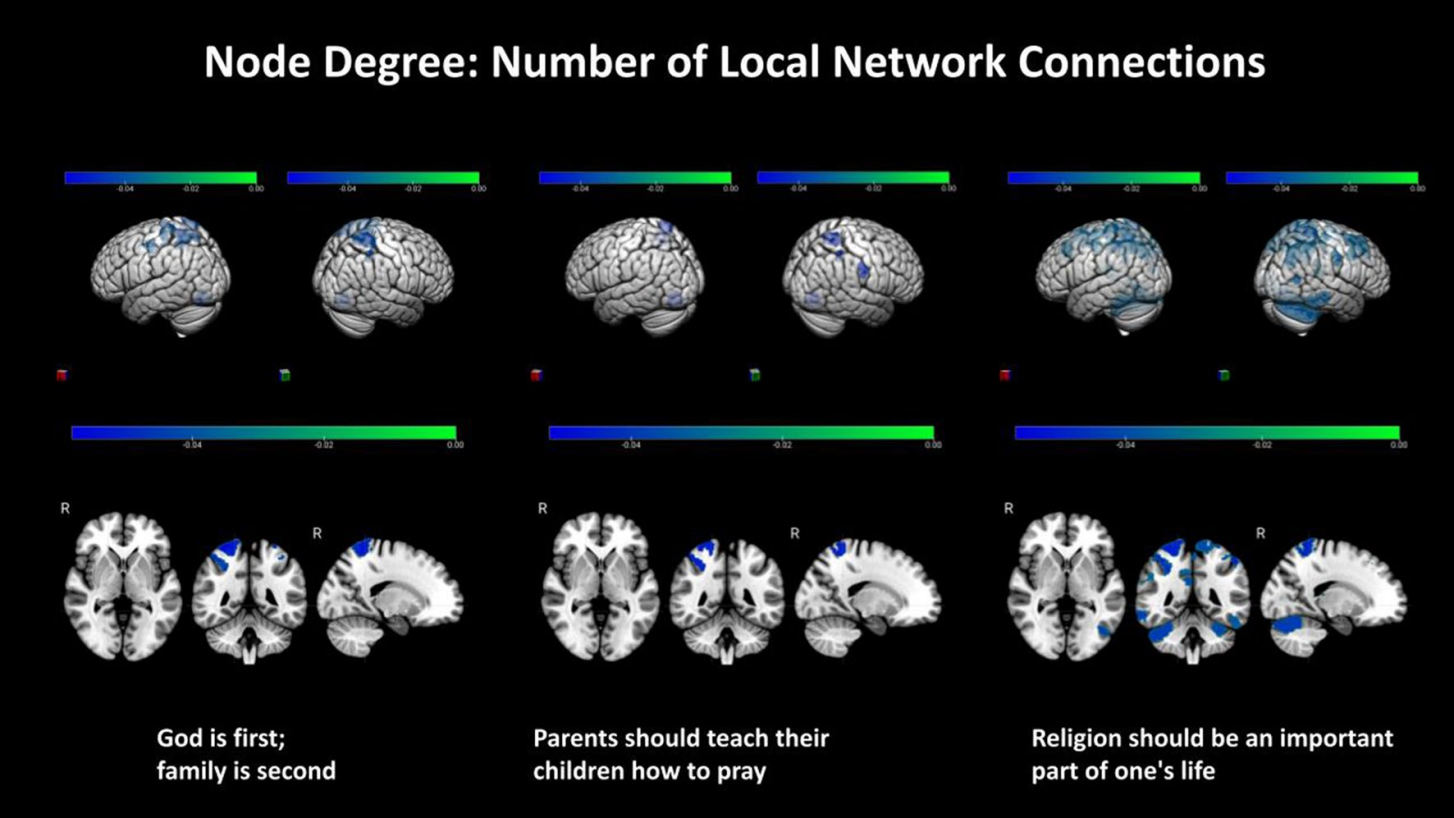
Significant negative associations were estimated, between strength of religious beliefs, including God is first, family is second, parents should teach their children how to pray, and religion should be an important part of one’s life and regional connectedness (degree). The colorbars represent the range of negative (green–blue) standardized regression coefficient values in statistical models that assessed these effects. Two and three-dimensional views of both hemispheres are shown.

The strength of the belief that ‘*God is first, family is second*’ was associated with lower node centrality in the left salience/ventral attention (p < 0.05), right somatomotor (p = 0.03) and parts of the right dorsal attention networks (p < 0.04), but with higher centrality bilaterally in the precuneus (p < 0.03). The strength of this belief was also associated with lower node degree in parts of the somatomotor and dorsal attention networks bilaterally (p < 0.05). The strength of the belief that ‘*parents should teach their children how to pray*’ was associated with lower centrality in parts of left salience, right somatomotor, right dorsal attention and right frontoparietal control networks (p < 0.04), but higher centrality bilaterally in the precuneus (p = 0.02). The strength of the belief that ‘*religion should be an important part of one’s life*’ was associated with lower degree in bilateral somatomotor, dorsal attention and frontoparietal control networks (p < 0.05), and the right DMN (p = 0.04). Finally, the strength of religious beliefs on the importance of God, religion, and prayer was negatively associated with local cerebellar properties (p < 0.01). Brain areas statistically associated with the strength of these beliefs are shown in Figs. 2 and 3. Effect sizes were overall small (Cohen’s d ~ 0.20 to ~ 0.29). Networks impacted locally by religious beliefs are summarized in Table S1.

#### Associations between religion-independent beliefs and brain network properties

The strength of the belief ‘*children should always do things to make their parents happy*’ was negatively correlated with whole-brain efficiency, robustness, and global clustering (p ≤ 0.03).

#### Individual networks and regional properties

The strength of only the belief ‘*children should always do things to make their parents happy*’ was inversely associated with lower topological efficiency, robustness, stability, global clustering and within network median connectivity of bilateral frontoparietal control and DM networks (p ≤ 0.04), left temporoparietal and reward networks (with the exception of non-significant within network connectivity in the latter and additional out-of-network connectivity in the former), right dorsal attention and right thalamus (p < 0.05). Effect sizes were small (Cohen’s d ~ 0.20 to ~ 0.38). These results are summarized in Table 7 and Table S1. The strength of this belief was also statistically associated with lower regional importance (node centrality) but not connectedness (p < 0.04) in the thalamus, caudate, and cerebellum. The impacted regions are shown in Fig. 2.

**Table 7:** Parameters of models assessing the relationship between parental belief that *‘children should always do things to make their parents happy’* and individual network properties. Across properties, effect sizes (Cohen’s d) were calculated with strength of belief = 1 (not at all) as the reference, with adjustments for unequal sample sizes. A range of effect sizes across properties, and 95% confidence intervals (CI) are provided.

Network	Model parameter	Efficiency	Global clustering	Median Conn (in)	Median Conn (out)	Robustness	Topological stability
**Belief: Children should always do things to make their parents happy**							
Left hemisphere							
Frontoparietal control	Beta	− 0.050	− 0.051	− 0.039	− 0.034	− 0.041	− 0.041
	p-value	0.004	0.004	0.019	0034	0.011	− 0.011
	Effect size (Cohen’s d)	0.215–0.343 (95% CI 0.142–0.473)					
Default mode	Beta	− 0.047	− 0.047	− 0.034	–	− 0.040	− 0.040
	p-value	0.010	0.010	0.042	–	0.018	0.018
	Effect size (Cohen’s d)	0.254–0.332 (95% CI: 0.159–0.462)					
Temporoparietal	Beta	− 0.046	− 0.047	− 0.032	− 0.038	− 0.045	− 0.045
	p-value	0.008	0.008	0.044	0.023	0.008	0.008
	Effect size (Cohen’s d)	0.224–0.251 (95% CI 0.111–0.381)					
Reward	Beta	− 0.032	− 0.032	–	–	− 0.032	− 0.032
	p-value	0.048	0.048	–	–	0.048	0.048
	Effect size (Cohen’s d)	0.202–0.226 (95% CI 0.101–0.356)					
Right hemisphere							
Dorsal attention	Beta	− 0.034	− 0.044	− 0.036	–	− 0.032	− 0.032
	p-value	0.046	0.036	0.046	–	0.046	0.046
	Effect size (Cohen’s d)	0.305–0.342 (95% CI 0.200–0.472)					
Frontoparietal control	Beta	− 0.050	− 0.044	− 0.035	–	− 0.042	− 0.042
	p-value	0.008	0.011	0.034	–	0.011	0.011
	Effect size (Cohen’s d)	0.276–0.377 (95% CI 0.213–0.474)					
Default mode	Beta	− 0.040	− 0.038	− 0.033	–	− 0.036	− 0.036
	p-value	0.039	0.039	0.044	–	0.039	0.039
	Effect size (Cohen’s d)	0.265–0.325 (95% CI 0.194–0.455)					
Thalamus	Beta	− 0.033	–	− 0.037	− 0.036	− 0.036	− 0.037
	p-value	0.042	–	0.039	0.039	0.039	0.039
	Effect size (Cohen’s d)	0.204–0.269 (95% CI 0.110–0.399)					

### Mediating and moderating relationships between parental beliefs, brain networks and cognitive performance

#### Mediating relationships

Mediation was investigated for identified significant relationships between parental beliefs and network properties (Path B), and between beliefs and task performance (Path A). Thus, relationships between Flanker, Card Sort, and Matrix Reasoning scores and topological properties of temporoparietal, frontoparietal control, DM, dorsal/ventral attention and reward networks and the thalamus were examined (Path C, Fig. 1a). Higher topological efficiency and global clustering in the right salience/ventral attention network, and higher efficiency, global clustering, within-network connectivity, robustness, and stability in the right frontoparietal control networks were associated with higher Flanker scores (p ≤ 0.04). Higher efficiency, global clustering, within-network median connectivity, robustness, and stability in the right temporoparietal, bilateral salience, dorsal attention, frontoparietal control, DMN, and reward networks, as well as median connectivity between the thalamus and the rest of the brain, and similarly for the right reward, control, and dorsal attention networks, was associated with higher Card Sort scores (p ≤ 0.04). Given significant direct relationships between multiple religious beliefs and performance in these two tasks, mediation was assessed only for them.

Full mediation models (Path D, Fig. 1a) assessed indirect effects of religious beliefs on Flanker and Card Sort scores through their impact on network topologies. The inverse relationship between strength of the belief ‘*God is first, family is second*’ and performance in the Flanker task was partially mediated by the belief’s effects on the properties of the right frontoparietal control network. Similarly, the inverse relationship between strength of the belief ‘*parents should teach their children how to pray*’ and performance in the Card Sort task was partially mediated by the belief’s effects on the properties of the right temporoparietal network. In both cases, mediating effects were small but significant (based on Sobel’s test, p < 0.02).

#### Moderating relationships

Moderation of the relationship between network topology and performance in the Flanker and Card Sort tasks by religious beliefs (Fig. 1b) was next investigated using simple slope analysis. The strength of the belief ‘God is first, family is second’ negatively moderated the positive relationship between the right frontoparietal control network and Flanker scores (p < 0.01). The effect size of this statistical moderation was small (f^2^ < 0.02). The strength of the belief that ‘*parents should teach their children how to pray*’ negatively moderated the positive relationship between the right temporoparietal network and performance in the Card Sort task (p ≤ 0.02). The effect size of the moderation was again small (f^2^ < 0.02). No other significant moderating effects were identified.

## Discussion

In this first-of-its-kind study we have investigated direct and indirect effects of parental religiosity (and for comparison effects of religion-independent beliefs on family values) on the topological organization of developing brain networks that support attention, executive function, cognitive flexibility, inhibitory control, and problem-solving. Although genetic factors play a critical role in the development of the brain’s neural circuits, environmental factors, and interactions between genes and environment may also contribute significantly to this process. An integral part of the youth environment is the family. In particular, parental beliefs and practices may have a significant impact on child development^62,63^. In this study we hypothesized that parental religiosity is an important aspect of these beliefs and practices and, as such, may significantly contribute to shaping developing children’s neural circuits. In our cohort, over 70% of participants had a religious affiliation, and their primary caregivers had at least moderately strong beliefs on the role of God, religion and prayer, which reflects the overall high religiosity of the US population^45^.

Stronger parental beliefs in the importance of God in one’s life, prayer and/or religion were associated with lower youth performance in the Flanker, Card Sort and Matrix Reasoning tasks. Lower performance in Matrix Reasoning and Card Sort tasks, which in part assess fluid reasoning and cognitive flexibility—both integral aspects of problem solving skills, may be linked to lower academic performance in subjects that depend on these skills, in agreement with prior reports on negative effects of religion on performance in mathematics and science^48,49^. These findings are also in agreement with prior work in a relatively large sample of adults (without neuroimaging), which has reported an inverse association between religiosity and cognitive flexibility^64^.

Despite a positive correlation between parental religious and non-religious beliefs possibly reflecting rigid parenting, the latter’s association with task performance was limited to the belief that children should do things to make their parents happy. The strength of this belief was associated with lower performance in Matrix Reasoning. Thus, some religious (thus context-specific) beliefs may specifically impact executive function, attention, and inhibitory control, but may affect other processes (e.g., fluid reasoning) in similar ways as religion-independent beliefs on family values^50^. The negative association between the strength of some religious beliefs and Flanker scores was somewhat surprising, given prior reports of positive correlations between religiosity (parent and/or youth), self-control and social skills in youth^48,65^. However, these relationships are complex and are affected by other parental attributes, such as parental education^66^. In our study, lower parental education and income correlated with stronger religious beliefs, which may partly explain the link with lower Flanker scores. Furthermore, increased externalizing behaviors, including impulsivity and lower self-control, have also been reported in adolescents whose parents are religious^50^.

Next, we examined the impact of religiosity on the topological organization of resting-state networks that support cognitive function across domains^67^. Many of these networks continue to develop during adolescence^68,69^, and their maturation can by profoundly affected by environmental factors. Stronger beliefs on the importance of God, religion, and prayer were associated with decreased efficiency, modular organization, topological robustness and stability, hub/local importance and connectedness of the temporoparietal, salience/ventral attention, dorsal attention, and frontoparietal networks. These changes were mostly lateralized, except in the temporoparietal network and the precuneus, which were bilaterally modulated.

The precuneus likely plays a central role in high-level cognitive processes, is considered a hub, i.e., a highly connected brain region where information is integrated^70^, is highly active at rest and may be involved in self-consciousness, self-related processing but also in episodic memory retrieval and response to pain^71^. At rest, it is functionally connected with the DMN, and during task performance it is coordinated with the left frontoparietal network^72^. In our cohort, higher topological importance of the precuneus was associated with stronger parental belief on the importance of God and religion, which may thus impact how youth perceive and/or feel about themselves. Prior work has reported aberrantly increased connectivity between the precuneus and other cortical areas in patients with major depressive disorder, possibly as the result of low self-esteem and changes in self-perception^73^. However, based on the low rates of major depressive disorder (2.0% past or present) and depressed mood (< 6.0%) in our cohort, it is unlikely that depressive symptoms are significant contributors to our findings. The precuneus and anterior frontal cortical regions are also part of the nonreward/punishment network^74^, which could be abnormally coordinated in children who may fear God’s punishment (although this belief was not measured in the MACV survey). Our study’s findings are, however, partly in agreement with prior work showing that God’s perceived level of involvement and beliefs related to the importance of religion are associated with positive and negative activation changes in the precuneus^75^.

The strength of multiple religious beliefs was negatively correlated with network-wide and local topological properties of the right frontoparietal control network, which continues to develop during adolescence and has been associated with executive control, internal representation of one’s physical self, illusion, and pain^75–78^. It is also synchronized with the DMN and the dorsal attention network during internally-focused cognitive processing. Stronger religious beliefs were negatively associated with both of these networks’ global and/or local topological properties. Frontoparietal and DMN topologies were also inversely modulated by the belief that children should always do things to make their parents happy. These results further suggest that early adolescents may partly perceive their parents’ religious beliefs as of rules on expected behavior, independently of spiritual content.

Global but not regional properties of the temporoparietal network, which is partially connected to the frontoparietal network and the DMN, were inversely associated with the strength of beliefs on the importance of God and prayer, and the belief that children should do things to make their parent happy (though only in the left hemisphere). Recent work has associated different subdivisions of this network with attention, episodic memory, empathy, social cognition, and the theory of mind (ToM)^79,80^. These subdivisions were not separately examined in this study, but of note is that the right temporoparietal network was primarily modulated by religious beliefs, whereas the left network was impacted by the religion-independent belief. Recent work has shown that the right temporoparietal network may be functionally separated into two parts, one related perhaps exclusively with social cognition and the other with both attention and ToM, and the left may play an important role in making inferences about someone else’s beliefs^81,82^. Thus, depending on their context, parental beliefs may differentially impact brain areas involved in belief perception and interpretation.

Topological properties of both dorsal and salience/ventral attention networks were inversely related with the strength of religious beliefs, and those of only the dorsal network with the strength of the belief that children should make their parents happy. One of the hubs of the salience network is the ACC^83,84^, which is involved in self-regulation, emotion, and social cognition^85^, and has been shown to be specifically modulated by religiosity/spirituality^7,20,37,86^. Mediation and prayer have been correlated with increased ACC activity^7,37,87^, and decreased reactivity in religious adults, which may help regulate anxiety^86^. However, our findings imply that early adolescents may interpret religious beliefs differently than adults, and the circuitry supporting selective attention and self-regulation may be adversely modulated by the misinterpretation of these beliefs. The strength of religious and non-religious beliefs was inversely associated with properties of the dorsal attention network, further supporting their common interpretation independently of context. However, regional properties of this network were affected only by religious beliefs on the importance of God, prayer, and religion. Prior studies comparing religious (Calvinists) and non-religious (atheist) young adults have shown that religiosity may bias visual attention. Our study suggests that parental beliefs may modulate youth brain circuits that support attention processes, which could lead to alterations in which aspects of sensory inputs they attend to^88,89^.

Regional properties of the cerebellum were also modulated by both religious beliefs (affecting regional importance and connectedness) and beliefs related to family values (affecting only regional importance). Multiple cognitive processes, beyond sensorimotor function, including visual working memory and attention tasks that are supported by the dorsal attention network, have also been associated with coordinated activity in the cerebellum^90,91^. Thus, decreased local properties of both the cerebellum and dorsal attention networks by parental beliefs may be linked to decreased performance in such tasks. Finally, topological properties of caudate and thalamus, which are central elements of the reward system^92,93^, were differentially modulated by the strength of the belief that children should do things to make their parents happy, but not by religious beliefs. Religion is associated with divine rewards but these are distant and abstract. In contrast, making parents happy may be associated with immediate and concrete rewards.

Finally, we examined indirect relationships between parental beliefs and youth task performance. The inverse relationship between strength of the belief God first, family second and Flanker scores was partially mediated by the adverse impact of this belief on topological properties of right frontoparietal network. Also, the negative association between the belief that parents should teach their children how to pray and Card Sort scores was partially mediated by the negative impact of this belief on properties of the right temporoparietal network. Given that this task measures executive function and cognitive flexibility and the temporoparietal network supports social cognition and ToM, among other functions, these results suggest that this belief may affect these processes through its impact on some of the circuits that support them. Our findings also suggest that parental religious beliefs may partially negatively moderate positive relationships between the topology of the frontoparietal network and Flanker scores, and that of the temporoparietal network and Cart Sort task scores. These findings point to complex direct and indirect relationships between parental religious beliefs and youth attention, executive function, cognitive flexibility and self-control.

Despite its strengths, including the large sample, assessments of both religious beliefs and practices, and network analyses across spatial scales, our study has some limitations. First, youth surveys on religiosity were not available at baseline in the ABCD study. Thus, the mediating effects of youth beliefs on the relationship between parental beliefs and network topology or cognitive task performance could not be assessed. Although secondary analyses were conducted using structural equation models that assumed youth religiosity (rather than individual beliefs) as a latent variable that could be estimated from available data, no significant mediating effects were estimated, likely due to the lack of explicit data on youth religious beliefs. In addition, the two questionnaires used in our analyses did not include more comprehensive and/or granular assessments of religiosity and/or parenting. Second, our study focused solely on resting-state networks and thus in the absence of tasks, and on cognitive task performance in the absence of neuroimaging data. However, resting-state networks represent the brain’s functional scaffolding, and their topological organization plays a fundamental role in cognitive function across domains. The identified mediating and moderating relationships also provide some evidence of the relationship between resting-state network activity and cognitive performance. Third, religious beliefs may be associated with other cultural and/or faith/denomination-specific factors not assessed in the ABCD study. In this study we have, however, assessed the effects of beliefs related to family values that could be at least partly culture-related. Finally, this is a retrospective analysis of a historically large sample that captures the heterogeneity of the early adolescent brain but was studied with a main goal independent of religiosity. Thus, there is a tradeoff between its limitations, including the instruments used to assess parent and/or youth religiosity, and a prospective investigation focusing exclusive on the neural basis of religiosity but in a much smaller sample that may not be representative of the general adolescent population.

This study makes a significant novel contribution and provides first insights into the effects of parental religiosity on developing neural circuits in early adolescence, a vulnerable period of profound brain changes^94–98^. It also highlights that in early adolescence, youth may interpret parental religious beliefs partly as rules reflecting rigid parenting, independently of their spiritual context. However, the religious content of some beliefs may also have distinct effects on the early adolescent brain, and may be associated with lower cognitive flexibility, attention, and inhibitory control and adversely impact the underlying functional circuitry. Our study also provides first evidence that multiple networks associated with self-referential processes and attention may be negatively affected by parental religious beliefs. These findings are in agreement with prior findings suggesting that religion may be a ‘mixed blessing’ for children^49^. However, the effects of parental beliefs on the developing brain may change over time, as parent–youth relationships evolve and the youth’s social environment grows. A future investigation of the longitudinal ABCD data could provide new insights into changes in youth perception and interpretation of religion, potentially leading to some positive effects of religiosity on brain and behavior.

## Methods

This study analyzed existing, anonymized and publicly available human data that are available through the National Institute of Mental Health’s repository (nda.nih.gov). The research was approved by the Institutional Review Board at Boston Children’s Hospital, and all analyses were performed in accordance with relevant guidelines and regulations.

### Participants

The ABCD is a historically large, nationwide study on adolescent brain development that studies 12,000 children (ages 9–10 at baseline) longitudinally^61^. Our cohort was selected based on availability and quality of the resting-state fMRI (data release 2.0.1), absence of clinical findings on structural MRI, and no history of bipolar disorder or ADHD, as both disorders that have been associated with aberrant functional connectivity^99,100^. The data quality criteria for inclusion are described in detail in^101,102^.

### Cultural surveys related to religion

Data on religious beliefs and practices were extracted from two surveys: the Mexican American Cultural Value Scale (MACV) modified from the scale developed by^103^, and the Parent Demographics Survey (PDEM). Both were completed by the primary caregivers. The MACV asked about parents’ attitudes towards religious and cultural statements, whereas the PDEM asked about their children’s religious beliefs and practices. The MACV survey was given to all participants’ caregivers independently of ethnicity or cultural heritage^104^. Seven questions related to religion were extracted: tell me how much you believe that (1) *one’s belief in God gives inner strength and meaning to life*; (2) *God is first; family is second*; (3) *parents should teach their children how to pray*; (4) *if everything is taken away, one still has their faith in God*; (5) *it is important to thank God every day for all one has*; (6) *it is important to follow the word of God*; (7) *religion should be an important part of one’s life*. Strength of belief was measured on a 5-point scale, from not at all (= 1) to completely (= 5). Corresponding data on youth religious and religion-independent beliefs were not available at the baseline assessment.

Three non-religious questions were also extracted from the MACV, assessing parental beliefs related to family values and children’s expected behavior towards the family: tell me how much you believe that (1) *children should always do things to make their parents happy*; (2) *children should be taught to always be good because they represent the family*; (3) *it is important to work hard and do one’s best because this work reflects on the family*.

Three questions were extracted from the PDEM: (1) *‘What is the child’s religious preference?’*, with choices for most common religious groups, and also atheist, agnostic and no particular preference. In analyses this was represented by a dichotomous variable: 1 = has religious preference, 0 = atheist, agnostic or no religion; (2) *‘How often does your child attend religious services?’*, measured on a 5-point scale: never (= 0) to more than once a week (= 4); (3) *‘How important are your child’s religious and spiritual beliefs in his/her daily life?’*, measured on a 4-point scale: not at all (= 1) to very much (= 4). Responses to these questions were available for > 95.0% of participants (Table 2b). Two additional questions on religious rules forbidding alcohol and drugs were missing responses for over half of the cohort and were not analyzed.

### Resting-State Functional Magnetic Resonance Imaging (rs-fMRI) analysis

The fMRI data processing is described in detail in^101,102^. Briefly, neuroimaging data minimally preprocessed by the Data Analysis, Informatics & Resource Center (DAIRC) of the ABCD study^105^ were further processed using our group’s Next Generation Neural Data Analysis platform (NGNDA)^106^. Each participant’s fMRI was coregistered to their structural MRI, slice-time and motion corrected, and normalized to MNI152 space. fMRI voxel time series were parcellated using 3 atlases (cortical, subcortical, and cerebellum) and individually denoised, resulting in 1088 signals with high signal-to-noise ratio^101^. Only runs with ≤ 10% frames censored for motion were considered for further analysis. Brain regions at rest are overall weakly synchronized, thus each participant’s run with the lowest overall median connectivity, typically coinciding with the run with the lowest number of censored frames, was included in the final analyses. These were conducted in the Harvard Medical School’s High-Performance Computing cluster, using the software MATLAB (Release 2021a; Mathworks, Inc).

Resting-state functional connectivity was estimated using signal cross-correlation and mutual information, to assess method dependence. Statistically similar correlation patterns were estimated by the two methods. Results based on peak cross-correlation between each pair of parcel BOLD signals are reported. Connectivity matrices were thresholded using the approaches described in^101^, using a relatively conservative population-based threshold, estimated as the upper 95% confidence interval of the moderately outlying correlation values (defined as median + 1.5*IQR), so that only moderate and high correlation values were included in the adjacency matrices used to calculate network properties.

Topological properties were estimated at three spatial scales: the entire brain, specific large-scale resting-state networks, and individual brain regions. Networks included those identified in^107^ and the reward network, encompassing dorsal prefrontal, orbitofrontal and anterior cingulate cortex, ventral striatum and pallidum, amygdala, thalamus, and hippocampus^108^. Algorithms from the Brain Connectivity Toolbox^109^ and the NGNDA platform were used in these estimations. Global properties included median connectivity, community structure (modularity and global clustering), global efficiency, small-worldness, topological robustness—measured by natural connectivity^110^, and topological stability—using the largest eigenvalue of the adjacency matrix^111^. Median connectivity was estimated both within each network and between nodes in and outside a network. Local properties included node eigenvalue centrality (regional topological importance), degree (number of node connections) and local clustering.

### Neurocognitive tasks

Age-adjusted and/or scaled scores in the Flanker^112^, Cash Choice^113^, Dimensional Change Card Sort^114^, and Matrix Reasoning^115^ tasks were analyzed. The Flanker task assesses executive function, attention, and inhibitory control. The Card Sort task measures executive function and cognitive flexibility. The Cash Choice task, measuring delay of gratification and impulsivity, asked participants a single question: “Would you rather have $75 in 3 days or $115 in 3 months?”. The Matrix Reasoning task measures fluid reasoning. The ABCD neurocognitive battery is described in detail in^60^. Tasks included in the battery were selected based on extensive prior work on performance reliability even in pre/early adolescence and into young adulthood. Data were available for > 98.0% of participants in all tasks [Flanker: n = 5497 (98.76%); Cash Choice: n = 5506 (98.92%); Dimensional Change Card Sort: 5501 (98.83%); Matrix Reasoning: 5467 (98.22%)].

### Statistical analysis

All analyses were based on statistical mediation and moderation ordinary linear regression models in Fig. 1a,b. The first set of models (Fig. 1a) assessed direct and indirect relationships between religiosity and cognitive task performance. The second set of models (Fig. 1b) assessed the moderating effects on religiosity on the relationship between connectome properties and task performance. First, the direct impact of strength of beliefs (primary predictors) on task scores (outcome) was assessed (Fig. 1a, Path A). Then the association between belief strength and network properties (the dependent variables) was assessed at each spatial scale (Path B). Given the large samples in these analyses, effect sizes were estimated using Cohen’s d, comparing each belief strength to the reference ‘not at all’ (= 1 in the scale). The relationships between network properties (primary predictors) and task scores were assessed in Path C. The full model (Path D) included both strength of beliefs and network properties (the mediators). Models assessing moderation of the relationship between network properties (predictor) and task scores (outcome) by parental beliefs (Fig. 1b), included belief strength as an additional independent variable and compared the relationship between predictor and outcome with vs without this variable, using a simple slope analysis. Given that strength of parental beliefs was measured on an ordinal scale, models assessing moderation assumed ‘not at all’ (= 1) in the scale as the reference and also included each strength level (2–5) as separate variables as well. Models also assessed the interactions between network properties (the primary predictors in the moderation models) and strength of beliefs.

Differences in imaging/population sampling sites were accounted for via propensity score adjustments. Models that included network measures were also adjusted for percent of frames censored for motion^101^. All models were adjusted for age, sex, race (dichotomized as white vs non-white, given the small samples of many racial categories and lack of statistical power to detect racial differences at a more granular level), ethnicity (Hispanic vs non-Hispanic), family income, BMI—since our prior work has identified negative associations between BMI and network properties^101^, importance of family togetherness (from the MACV survey) and family size (from the PEDM survey). All p-values were adjusted for the False Discovery Rate (FDR), using well-established approaches, depending on the assumption of variable (and p-value) independence^116^ or dependence^117^, given potentially correlated network properties. When sets of p-values are reported in text, the largest significant value is reported, indicating that all others are smaller or equal to that and similarly, for nonsignificant p-values, the smallest nonsignificant value is reported. Collinearity between independent variables was assessed before augmenting models, and multiple strategies were used to identify a parsimonious set of independent variables. The results reported here are from models that included a common fixed number of parameters, so that Paths A-D are comparable. Model fit was assessed using the adjusted R^2^ and Akaike information criterion (AIC) estimators. All figures based on the results of the statistical models were generated using the software MRICroGL (NITRC.org).

Given the lack of survey data on youth beliefs, which could potentially mediate the relationship between parental beliefs and topological network parameters, a secondary analysis using a structural equation modeling (path analysis) framework was conducted. The models assumed youth religiosity (rather than individual beliefs) as an aggregate latent variable, which could be estimated from the only available data related to youth religiosity, namely frequency of religious service attendance and importance of religion in the youth’s daily life, both of which were reported by the parents.

#### Split-sample model validation

In addition to developed statistical models based on the entire cohort, an out-of-sample approach was also used for validation. Seventy-five percent of participants (n = 4175) were randomly selected to develop models, and the remaining 25% were used to validate them. The process was repeated 100 times. In addition to the AIC, the coefficient of variation of the root mean-squared error [CV(RMSE)], between predictions and validation data, was used as the measure of the model’s predictive power.

## Supplementary Information

Supplementary Information.

## Data Availability

The data are already publicly available through the NIMH repository NDA (nda.nih.gov). Assession number (DOI) associated with study in NDA repository: https://www.doi.org/10.15154/1526322.
