# Supplementary material for: Parental religiosity is associated with changes in youth functional network organization and cognitive performance in early adolescence
Source: Sci Rep. 2022 Oct 15;12:17305. doi: 10.1038/s41598-022-22299-6 (PMC9569366; doi:10.1038/s41598-022-22299-6)
Supplement: Supplementary file 1 — Supplementary Information. [file 41598_2022_22299_MOESM1_ESM.pdf]

SUPPLEMENTAL MATERIAL

Table S1: Cognitive tasks and resting-state networks and brain regions significantly impacted by individual religion-dependent and independent beliefs. Direction of association is indicated as +/-. Other refers to Default mode and reward networks and the thalamus, which were topologically impacted only by one religion-independent belief.

Belief	Cognitive task			Global topological properties					Local topological properties						
	Flanker	Card Sort	Matrix Reasoning	Temporoparietal	Frontoparietal Control	Dorsal Attention	Salience/Ventral Attention	Other*	Dorsal Attention	Frontoparietal Control	Thalamus	Precuneus	Default Mode	Salience/Ventral Attention	Somatomotor
**Religious beliefs**															
**God is first, family is second**	✓(-)		✓(-)	✓(-)	✓(-)	✓(-)	✓(-)		✓(-)	✓(-)		✓(+)		✓(-)	✓(-)
**Parents should teach their children how to pray**	✓(-)	✓(-)	✓(-)	✓(-)					✓(-)	✓(-)		✓(+)		✓(-)	✓(-)
**If everything is taken away, one still has their faith in God**	✓(-)	✓(-)	✓(-)						✓(-)						
**It is important to thank God every day**	✓(-)		✓(-)						✓(-)						
**It is important to follow the word of God**	✓(-)		✓(-)												
**Religion should be an important part of one's life**	✓(-)		✓(-)			✓(-)			✓(-)	✓(-)			✓(-)		✓(-)
**God gives inner strength and meaning to life**			✓(-)												
**Religion-independent beliefs**															
**Children should always do things to make their parents happy**			✓(-)	✓(-)	✓(-)	✓(-)		✓(-)			✓(-)				

Supplemental description of cognitive tasks and list of brain networks analyzed in the study

1. Cognitive Tasks

a. NIH Toolbox Flanker: Executive Function; Inhibitory Control; Attention: The ABCD study used a variation of the Eriksen Flanker task (Eriksen, 1974), to assess ability to suppress responses to surrounding stimuli (flankers) that are congruent and incongruent with the target. Trial blocks with arrows stimuli were used in study. Flanking stimuli were facing in the same or opposite direction as the target arrow. Participants were instructed to push a button to indicate whether the target was facing right or left. Scores were based on speed and accuracy. More details on task specifics used in the ABCD study are provided in (Luciana, 2018).

b. NIH Toolbox Dimensional Change Card Sort: Executive Function; Cognitive Flexibility: Participants were required to sort a series of objects by color or shape. They completed 3 blocks of trials in which they sorted objects by one dimension, then the other, and finally switched from one to the other in a pseudo-random order. They were shown 2 objects at the bottom of a screen and were asked to sort a third one (presented in the middle of the screen) by shape or color, so that it matched one of the two objects (Zelazo, 2006). Scores were based on accuracy and reaction time. More details on the task variant used in the ABCD study are provided in (Luciana, 2018).

c. Matrix Reasoning Task: Fluid Reasoning; Visuospatial ability; Part-whole reasoning; Visual sequencing: The automated version of this test, which is part of the Wechsler Intelligence Test for Children-V (WISC-V: Wechsler, 2014), was used in the ABCD study. Examiners presented participants with an incomplete visuospatial series of stimuli, and participants were asked to select 1 of 4 stimuli to complete the series. The task ended when the participant failed to correctly complete the series on 3 consecutive trials. when the participant fails three consecutive items. Standardized scores were estimated from raw scores and a normative database (mean score = 10.0, SD = 3.0). Additional details on this task can be found in (Luciana, 2018).

d. Cash Choice Task: Delay of gratification; Motivation; Impulsivity: This was a one-item task (Wulfert, 2002). Participants were asked: ‘Let’s pretend a kind person wanted to give you some money. Would you rather have $75 in three days or $115 in 3 months?’. Response options were yes, no or ‘can’t decide’.

References

1. Luciana, M. *et al.* Adolescent neurocognitive development and impacts of substance use: Overview of the adolescent brain cognitive development (ABCD) baseline neurocognition battery. *Dev. Cogn. Neurosci.* **32**, 67–79 (2018).

2. Eriksen, B. A. & Eriksen, C. W. Effects of noise letters upon the identification of a target letter in a nonsearch task. *Percept. Psychophys.* **16**, 143–149 (1974).

3. Zelazo, P. D. The Dimensional Change Card Sort (DCCS): A method of assessing executive function in children. *Nat. Protoc.* **1**, 297–301 (2006).

4. Wulfert, E., Block, J. A., Santa Ana, E., Rodriguez, M. L. & Colsman, M. Delay of gratification: Impulsive choices and problem behaviors in early and late adolescence. *J. Pers.* **70**, 533–552 (2002).

5. Wechsler, D. & Kodama, H. *Wechsler intelligence scale for children*. vol. 1 (Psychological corporation New York, 1949).

2. Resting-state networks

The networks analyzed in this study included the 17 networks identified in (Yeo, 2011), the thalamus, cerebellum, amygdala and basal ganglia networks identified using subcortical (Melbourne; Tian, 2020) and cerebellar (Diedrichsen, 2009) atlases, and the reward network, which included parts of the dorsal attention (frontal eye fields), salience network (prefrontal cortex), limbic network (orbitofrontal cortex and amygdala), control network (medial prefrontal cortex), default mode network (dorsolateral and medial prefrontal conrtex), hippocampus, thalamus, amygdala and parts of the basal ganglia (Schultz,

2000). The 17-network estimate described in Yeo, 2011 represented a fractionation of a 7-network estimate that included visual, somatomotor, dorsal attention, salience/ventral attention, default mode, frontoparietal control and limbic networks. The fractionation, which resulted in subnetworks of the visual network and the emergence of additional temporo-parietal networks is described in detail in Yeo, 2011.

References

1. Yeo BTT, Krienen FM, Sepulcre J (2011), The organization of the human cerebral cortex estimated by intrinsic functional connectivity, J Neurophysiol, 106: 1125–1165.

2. Schultz W, Tremblay L, Hollerman JR. 2000. Reward Processing in Primate Orbitofrontal Cortex and Basal Ganglia. Cer Cortex. 10(3):272-283.
